# Normal Distal Tibiofibular Syndesmosis Assessment Using Postmortem Computed Tomography (PMCT)

**DOI:** 10.3390/diagnostics14010036

**Published:** 2023-12-24

**Authors:** Jahyung Kim, Jeong-Hyun Park, Hyung-Wook Kwon, Mijeong Lee, Digud Kim, Yu-Jin Choi, Kwang-Rak Park, Sookyoung Lee, Jaeho Cho

**Affiliations:** 1 Department of Orthopaedic Surgery, Armed Forces Yangju Hospital, Yangju 11428, Republic of Korea; hpsyndrome@naver.com; 2 Department of Anatomy & Cell Biology, School of Medicine, Kangwon National University, Chuncheon 24341, Republic of Korea; jhpark@kangwon.ac.kr (J.-H.P.); kwenhw@naver.com (H.-W.K.); toff337@hanmail.net (M.L.); oe5235@naver.com (D.K.); police5565@hanmail.net (Y.-J.C.); 3 Department of Anatomy, College of Korean Medicine, Sangji University, Wonju 26339, Republic of Korea; airboba@naver.com; 4 Division of Forensic Medical Examination, National Forensic Service, 10 Ipchun-ro, Wonju 26460, Republic of Korea; heart@korea.kr; 5 Department of Orthopaedic Surgery, Chuncheon Sacred Heart Hospital, Hallym University, Chuncheon 24253, Republic of Korea

**Keywords:** distal tibiofibular relationship, syndesmosis, postmortem, computed tomography

## Abstract

Background: Distal tibiofibular syndesmotic injuries, often misdiagnosed, can lead to substantial morbidity. This study utilized postmortem computed tomography (PMCT) to define normal syndesmotic relationships in 131 subjects. Methods: Three parameters were measured: fibular rotation (FR), sagittal translation (ST), and incisura depth (ID). Results: Interobserver reliability was excellent for FR and ID but moderate for ST. Anatomical variability was wide, with FR ranging from −0.4° to 16.6°, ST from 0.33 mm to 3.49 mm, and ID from 1.89 mm to 6.05 mm. Side-to-side variability within subjects was minimal. Gender-specific differences were observed in ST, possibly due to size variations, highlighting the need for gender-specific diagnostic criteria. Conclusions: Although establishing universal reference values is challenging, using contralateral ankles for comparison can enhance diagnostic accuracy in syndesmotic injuries. This study, the first of its kind, offers valuable insights into normal distal tibiofibular syndesmotic relationships based on PMCT data. Future validation studies in patients with syndesmotic injuries can further improve diagnostic accuracy.

## 1. Introduction

In the anatomical literature, the connection between the distal tibia and the fibula that creates a mortise for the trochlea of the talus is referred to as a syndesmosis [1]. Clinically, injuries to syndesmosis are known to occur in 1–18% of all ankle sprains, while the actual frequency might be higher due to misdiagnosis in those with subtle injuries [2]. Moreover, syndesmotic injury was seen in 44% of the 266 individuals with acute ankle fractures who underwent arthroscopic diagnosis [3]. Since restoration of stability and consistency of the mortise in ankle injuries is one of the key prerequisites for favorable long-term functional outcomes, misdiagnosis or inaccurate reduction in syndesmotic injury can result in substantial morbidity [4]. Therefore, researchers have discovered a variety of clinical examinations and radiographic methods in an effort to enhance diagnostic accuracy of syndesmotic injuries.

Of the many radiographic modalities to evaluate syndesmosis status, computed tomography (CT) is considered to have high sensitivity and specificity [2]. To be specific, studies have suggested multiple radiographic parameters on the axial CT scans which can help effectively predict distal tibiofibular syndesmotic injuries [5,6,7]. However, definite reference figures of the measurements along with permissible ranges indicating normal distal TFS could not be specified due to significant anatomic variation between individuals. Rather, researchers recommend the use of a patient’s contralateral ankle for comparison [8,9]. In addition, quantitative evaluation for normal tibiofibular relationships using CT in large numbers of living subjects is ethically limited. 

Postmortem computed tomography (PMCT) is used in the context of trauma either as one of the pre-autopsy measures or as a part of a complementary virtual autopsy protocol [10]. It has been shown to guide identifying the occult cause of death along with autopsy, therefore improving the completeness of trauma registries [11]. In fact, PMCTs of bilateral ankles are routinely performed for all the subjects at the National Forensic Service (NFS) in South Korea, which may provide a vast amount of information in terms of normal radiographic findings of the ankle joint without ethical limitations. The objective of this study was to define the normal distal tibiofibular syndesmotic relationship of an uninjured ankle using PMCT among large subjects and to report the reliability of the measurements. We hypothesized that reference values and ranges indicating normal distal tibiofibular syndesmosis could be established using PMCT.

## 2. Materials and Methods

### 2.1. Study Design

This study protocol was reviewed by the institutional review board of our institution and informed consent was waived by the board. The study design of the previously reported studies was adopted as a reference for this study [8,9,12]. We randomly selected 200 subjects, which was twice the size of the samples in previously reported studies, who underwent bilateral ankle CT provided by NFS in South Korea. Subjects with histories or signs of ankle fractures, chronic ankle instability, ankle osteoarthritis, or apparent scars of previous ankle surgery were excluded. Those without symmetric bilateral axial CT images due to inappropriate posture, traumatic amputation, or rigor mortis were also omitted. Ultimately, 131 subjects were selected for analysis. The subjects consisted of 66 males and 65 females. The age of death was 53.62 ± 1.91 (range 21 to 89).

### 2.2. Measurements 

**Figure 1:**
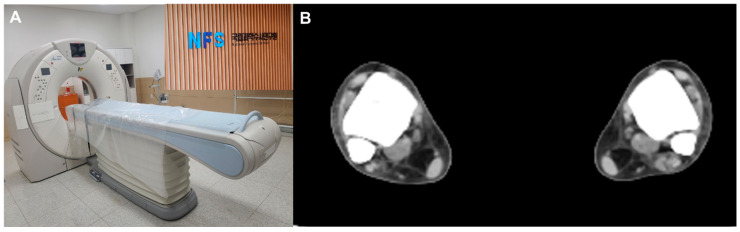
(**A**) All subjects were screened postmortem computed tomography, (**B**) A single axial image 1 cm above the tibial plafond, a level where the length of the distal anterior tibial tubercle is the greatest, was selected for measurement.

On arrival at NFC, all subjects were screened with bilateral ankle CTs (Aquilion PRIME TSX-303A, CANON Medical Systems Corporation, Tokyo, Japan) (Figure 1A). To provide consistent axial slices parallel to the plafond, the vertical axis of the tibia and the horizontal axis of the plafond were used as reference axes. In addition, the bimalleolar axis, a line perpendicular to the line connecting anterior aspects of the medial and lateral malleoli, was used as a reference to guarantee neutral rotation [13,14]. For each CT scan, a single axial image 1 cm above the tibial plafond, a level where the length of the distal anterior tibial tubercle is the greatest, was selected for measurement [13] (Figure 1B).

**Figure 2:**
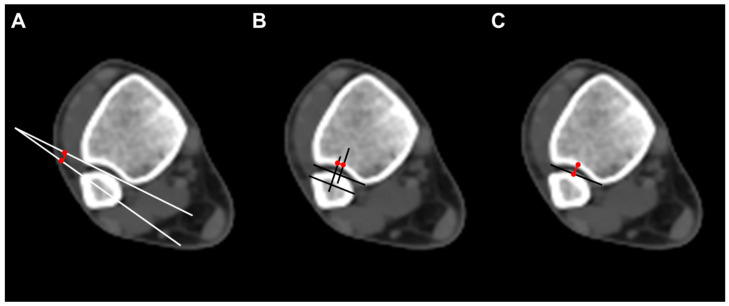
Radiographic measurements. (**A**) Fibular rotation (angle between a line drawn between the anterior and posterior borders of the incisura and the fibular orientation line); (**B**) sagittal translation (difference between the midpoint of the incisura length and the midpoint of the fibular length line. The fibular length line was drawn from the most anterior point of the fibula, parallel to the incisura length); (**C**) incisura depth (distance from the tibial cortex at the midpoint of the incisura to the line that joins the most anterior point with the most posterior point of the incisura).

To evaluate the anatomical distal tibiofibular relationship, we measured three parameters on axial CT scan images. Fibular rotation (FR) was defined as the angle between a line drawn between the anterior and posterior borders of the incisura and the line through the anterior and posterior fibular tubercles. The angle was considered positive if the fibula was internally rotated compared with the incisura [12]. Sagittal translation (ST) of the fibula was measured as the difference between the midpoint of the incisura length and the midpoint of the fibular length line. The fibular length line was drawn from the most anterior point of the fibula, parallel to the incisura length [15]. Incisura depth (ID), which means the distance from the tibial cortex at the midpoint of the incisura to the line that joins the most anterior point with the most posterior point of the incisura, was also measured (Figure 2). 

### 2.3. Statistical Analysis

The intraclass correlation coefficient (ICC) was used to calculate inter- and intra-observer reliabilities for all measurements. According to the definition of Landis and Koch, ICCs of 0.81 to 1.00, 0.61 to 0.80, 0.41 to 0.60, 0.21 to 0.40, and 0.00 to 0.20 were interpreted as excellent, good, moderate, fair, and poor, respectively. Measurements were summarized with means, standard deviations, and ranges. These were sorted in terms of gender, and independent t-tests were used to compare each measured parameter between males and females. A *p* < 0.05 was considered to indicate a significant difference. The variance between the right and left ankles of each subject was calculated. The maximum expected variance between the ankles of an uninjured individual was calculated based on 95% confidence intervals. Data were analyzed using IBM SPSS Statistics version 23.0 for Windows (IBM Co., Armonk, NY, USA).

## 3. Results

The fibular rotation (FR) and incisura depth (ID) demonstrated excellent and good interobserver reliability, respectively (0.7537 and 0,8765), but sagittal translation (ST) showed moderate agreement (0.4285) (Table 1). 

The mean FR measured was 8.14 ± 3.22° (−0.4 to 16.6). Also, the mean ST and ID were 1.33 ± 0.54 mm (0.33 to 3.49) and 3.78 ± 0.82 mm (1.89 to 6.05), respectively. There was significant anatomic variability between individuals. In terms of gender differences, statistically significant gender differences were discovered in ST (*p* < 0.001). However, no significant difference between males and females was observed in FR and ID (Table 2). 

The average measurement variance between the right and left ankles of each subject was determined. The FR did not vary by more than 0.57°, the ST did not vary by more than 0.26 mm, and the ID did not vary by more than 0.32 mm between ankles of the same subject (Table 3).

## 4. Discussion

PMCT is a fast and cost-effective modality for evaluating the body and the cause of death in a non-invasive manner [16]. Although conventional autopsy remains the gold standard, its steady decline due to personal or religious reasons has led to an increased interest in PMCT [10]. In fact, PMCT is known to be more effective than autopsy in detecting bony injuries, fluid in airways, gas in internal organs, major hemorrhages, fatty liver, stones, and bullet fragments [17]. As a result, PMCT is routinely being used for screening purposes at NFS in Korea.

PMCT plays a significant role in clinical medicine, particularly in forensic pathology and postmortem investigations. First, PMCT is extensively used in forensic investigations to assist in determining the cause of death, identifying injuries, and reconstructing events leading to death. It provides detailed information about internal injuries, fractures, gunshot wounds, and other trauma that may not be apparent externally. It aids in the identification of potential criminal activity, contributes to forensic analysis, and assists in providing evidence in legal proceedings. Second, virtopsy, or virtual autopsy, is a non-invasive approach that combines PMCT imaging with other imaging modalities, such as MRI and 3D surface scanning. It enables a comprehensive examination of the deceased’s body, providing information about injuries, organ pathologies, and anatomical variations. Virtopsy can be particularly valuable when traditional autopsies are not possible or when families prefer non-invasive methods. Third, PMCT serves as a valuable tool for documentation and record-keeping. It generates high-resolution images of the body, allowing for accurate documentation of injuries, pathological findings, and anatomical structures. These records can be stored digitally and accessed for future reference or research purposes. Fourth, PMCT can be used as an educational tool for medical students, residents, and other healthcare professionals. It allows for the study of normal and pathological anatomy, enhancing understanding and providing a visual representation of internal structures. It can also aid in surgical planning and simulation, providing valuable insights into the patient’s anatomy before performing procedures. Lastly, PMCT contributes to anatomical research by providing detailed imaging data of human cadavers. Researchers can study anatomical variations, evaluate the effects of diseases or injuries, and investigate the anatomical relationships between structures. This research aids in advancing medical knowledge, improving surgical techniques, and enhancing understanding of human anatomy. Additionally, PMCT serves as a quality assurance tool in clinical practice. It allows for retrospective analysis and review of imaging findings, contributing to continuous learning and improvement in radiological interpretations. It provides an opportunity to identify missed or misinterpreted findings, enhancing the accuracy and reliability of postmortem imaging. Therefore, the authors judged that PMCT was clearly important data for anatomical research related to clinical practice, although it is important to note that PMCT is primarily utilized in the postmortem setting and differs from clinical CT imaging performed on living patients. The utilization of PMCT in this study provided a unique opportunity to define the normal syndesmotic relationships in a substantial cohort of 131 subjects.

In general, the syndesmosis (distal tibiofibular relationship) morphology refers to the anatomical structure and characteristics of the syndesmosis joint, which is a fibrous joint found between certain bones, such as the tibia and fibula in the lower leg. The specific morphology of the syndesmosis joint can vary between individuals due to natural anatomical variations [1]. While there are general patterns and typical features of the syndesmosis joint, it is important to note that the exact morphology of the syndesmosis can differ from person to person. Factors such as bone shape, size, and orientation can contribute to anatomical variations in syndesmosis. Additionally, injuries or pathological conditions can affect the morphology of syndesmosis. For example, a sprain or disruption of syndesmosis can lead to instability and changes in the joint’s morphology. Therefore, it is not accurate to assume that the syndesmosis morphology would be the same in the same specimen or between different individuals. Variations are expected, and it is important to consider individual anatomical differences when assessing the morphology of the syndesmosis joint [2,8]. 

This study intended to provide a normal range of normal tibiofibular relationships at the syndesmosis made on axial PMCT images. However, significant variability of measurements existed between individuals due to anatomic diversity between individuals. FD ranged from −0.4 to 16.6 degrees. Similarly, the mean ST and ID showed wide ranges of 0.33 to 3.49 and 1.89 to 6.05, respectively. Instead, maximal side-to-side variability within each subject for FD, ST, and ID was 0.57°, 0.26 mm, and 0.32 mm. As a result, rather than relying on the normal reference figures of each ankle, using the subject’s contralateral ankle for comparison would facilitate an accurate assessment of syndesmotic structures. In other words, we suggest taking bilateral ankle CT for comparison in case a syndesmotic injury is suspected.

In this study, significant gender-specific differences were discovered in CT measurements of ST. An increased ST was seen in male objects, although interobserver its correlation was lower than other radiographic parameters. Previous studies also reported similar results and interpreted it as a size difference between genders [8,9]. To eliminate the effect of the subject’s size, Dikos et al. re-expressed the radiographic parameters into ratios, which showed no significant gender differences [8]. Since currently accepted radiographic parameters that are expressed in numbers do not take gender differences into consideration, gender-specific diagnostic criteria would have to be established to evaluate the syndesmosis adequately.

The main limitation of this study is that we relied only on histories, signs, or previous operative scars to confirm that subjects had an uninjured syndesmosis. Because this was a retrospective study and subjects were not living objects, a detailed history or physical examination was not available. Further imaging studies like MRI or ultrasonographic imaging might have helped exclude subjects without intact syndesmotic structures. The sample size was not calculated in this study either. We hope that this study could be used as a pilot study to organize a larger scale analysis based on accurate sample size calculation. In addition, the subject’s size was not addressed because the condition of the body was not suitable for measurement on arrival at NFS in most cases. Information regarding the height or weight of the subjects in this study could have raised the consistency of the included cohort and made the measurements more easily applicable to the general population. Nevertheless, we believe that the present study is valuable in respect of it being the first to use PMCT to define normal tibiofibular syndesmotic relationships among the largest number of subjects. If possible, a validation study based on the result of this study on patients with syndesmotic injuries would be worthwhile in terms of enhancing the diagnostic accuracy of CT. 

## 5. Conclusions

Due to wide anatomical variability between individuals, establishing reference values and ranges indicating normal distal tibiofibular syndesmosis may not be possible. Instead, using the patient’s contralateral ankle for comparison provides a more precise definition of the normal relationship and would be helpful for the diagnosis of occult syndesmotic injuries. Moreover, we believe that PMCT could be used as an effective diagnostic tool to determine normal anatomical measurements or variations in the field of orthopedic surgery.

## Figures and Tables

**Table 1:** Interobserver agreement.

	Rater 1	Rater 2	ICC		
	Mean ± SD	Mean ± SD	Estimate	Lower	Upper
Fibular rotation (°)	8.33 ± 3.43	7.96 ± 3.74	0.7537	0.6525	0.8254
Sagittal translation (mm)	1.53 ± 0.45	1.13 ± 0.82	0.4285	0.0886	0.629
Incisura depth (mm)	3.76 ± 0.83	3.81 ± 0.91	0.8765	0.8256	0.9125

SD: standard deviation, ICC: Intraclass correlation coefficient, mm: millimeter, **°**: degree.

**Table 2:** Measurements of the anatomical distal tibiofibular relationship.

	Total (*N* = 131)			Male (*N* = 66)			Female (*N* = 65)			*p*-Value
	Mean ± SD	Median (IQR)	Range (Min to Max)	Mean ± SD	Median (IQR)	Range (Min to Max)	Mean ± SD	Median (IQR)	Range (Min to Max)	
Fibular rotation (°)	8.14 ± 3.22	7.83 (6.12, 10.5)	−0.4 to 16.6	8.43 ± 3.24	8.05 (6.76, 10.91)	−0.4 to 16.6	7.85 ± 3.2	7.38 (5.22, 10.43)	2.3 to 15.43	0.3003
Sagittal translation (mm)	1.33 ± 0.54	1.27 (0.94, 1.56)	0.33 to 3.49	1.54 ± 0.6	1.47 (1.15, 1.73)	0.33 to 3.49	1.11 ± 0.35	1.02 (0.89, 1.29)	0.61 to 2.46	<0.001
Incisura depth (mm)	3.78 ± 0.82	3.75 (3.21, 4.42)	1.89 to 6.05	3.77 ± 0.95	3.73 (3, 4.49)	1.89 to 6.05	3.8 ± 0.67	3.77 (3.38, 4.24)	2.49 to 5.12	0.8311

SD: standard deviation, IQR: interquartile range, mm: millimeter, **°**: degree.

**Table 3:** Side-to-side variability.

	Mean ± SD	Range (Min to Max)	Maximum Variability
Fibular rotation (°)	−1.12 ± 3.2	−10.45 to 7.95	−0.57
Sagittal translation (mm)	0.14 ± 0.69	−2.31 to 2.18	0.26
Incisura depth (mm)	0.18 ± 0.81	−2.17 to 2.64	0.32

SD: standard deviation, mm: millimeter, **°**: degree.

## Data Availability

The data used to support the findings of this study are available from the corresponding author upon request.
